# RNAi and Homologous Over-Expression Based Functional Approaches Reveal Triterpenoid Synthase Gene-Cycloartenol Synthase Is Involved in Downstream Withanolide Biosynthesis in *Withania somnifera*

**DOI:** 10.1371/journal.pone.0149691

**Published:** 2016-02-26

**Authors:** Smrati Mishra, Shilpi Bansal, Bhawana Mishra, Rajender Singh Sangwan, Asha, Jyoti Singh Jadaun, Neelam S. Sangwan

**Affiliations:** 1 Department of Metabolic and Structural Biology, CSIR-Central Institute of Medicinal and Aromatic Plants (CSIR-CIMAP), P.O. CIMAP, Lucknow, India; 2 Center of Innovative and Applied Bioprocessing (CIAB) (A National Institute under Department of Biotechnology, Government of India), C-127, Phase-8, Industrial Area, S.A.S. Nagar, Mohali, Punjab, India; Instituto de Biología Molecular y Celular de Plantas (IBMCP), SPAIN

## Abstract

*Withania somnifera* Dunal, is one of the most commonly used medicinal plant in Ayurvedic and indigenous medicine traditionally owing to its therapeutic potential, because of major chemical constituents, withanolides. Withanolide biosynthesis requires the activities of several enzymes *in vivo*. Cycloartenol synthase (*CAS*) is an important enzyme in the withanolide biosynthetic pathway, catalyzing cyclization of 2, 3 oxidosqualene into cycloartenol. In the present study, we have cloned full-length *WsCAS* from *Withania somnifera* by homology-based PCR method. For gene function investigation, we constructed three RNAi gene-silencing constructs in backbone of RNAi vector pGSA and a full-length over-expression construct. These constructs were transformed in *Agrobacterium* strain GV3101 for plant transformation in *W*. *somnifera*. Molecular and metabolite analysis was performed in putative *Withania* transformants. The PCR and Southern blot results showed the genomic integration of these RNAi and overexpression construct(s) in *Withania* genome. The qRT-PCR analysis showed that the expression of *WsCAS* gene was considerably downregulated in stable transgenic silenced *Withania* lines compared with the non-transformed control and HPLC analysis showed that withanolide content was greatly reduced in silenced lines. Transgenic plants over expressing *CAS* gene displayed enhanced level of *CAS* transcript and withanolide content compared to non-transformed controls. This work is the first full proof report of functional validation of any metabolic pathway gene in *W*. *somnifera* at whole plant level as per our knowledge and it will be further useful to understand the regulatory role of different genes involved in the biosynthesis of withanolides.

## Introduction

*Withania somnifera* (Indian ginseng) is one of the most famous and widely used medicinal plants [1]. *W*. *somnifera*, commonly known as Ashwagandha, is an ancient medicinal herb that has been used in various pharmacological preparations traditionally. The main constituents for its pharmacological activities are withanolides [2].Withanolides has been shown to exert many pharmacological effects, including immune system modulation, antistress activities, antihyperglycemic activities, anti-inflammatory, antioxidant, and anti-cancer effects [3–6]. Withanolides are basically of the terpenoid origin and are one of the the largest group of natural products with diverse molecular structures. Withanolides or 22-hydroxy ergostane-26-oic acid 26, 22-δ-lactones are C-28 steroidal lactones based on ergostane framework which when oxidized at C-22 and C-26 form a δ-lactone ring [7]. Till now several withanolides have been isolated from different parts of *W*. *somnifera* including major withanolides i.e. withaferin A, withanone and withanolide A, withanolide D etc [8]. Withanolides are synthesized through isoprenoid pathway *via* both mevalonate and non-mevalonate pathways [9]. The first committed step in the triterpenoid biosynthesis occurs by cyclization of epoxysqualene, leading to the formation of C_30_H_50_O product with 1–5 rings [10]. Plants contain different oxidosqualene cyclases (OSCs) for a single substrate to synthesize different set of sterols and other triterpenoids [11].

**Fig 1:**
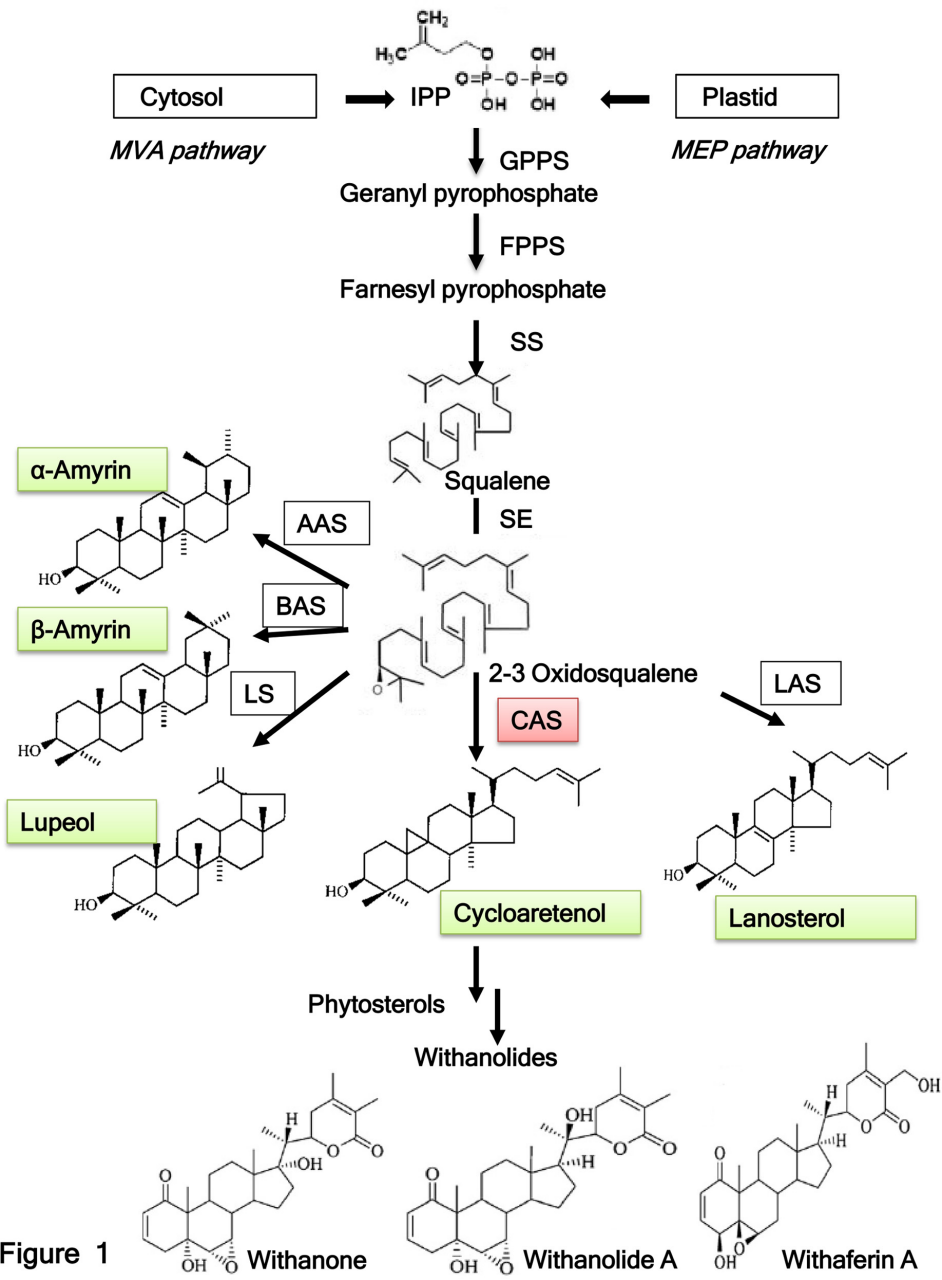
Withanolide biosynthetic pathway. IPP, isopentenyl pyrophosphate; MVA, mevalonate; MEP, 2-C-methyl-D-erythritol 4-phosphate GPPS, geranyl pyrophosphate synthase; FPPS, farnasyl pyrophosphate synthase; SS, squalene synthase; SE squalene epoxidase; CAS, cycloartenol synthase; LAS, lanosterol synthase; LS, lupeol synthase; BAS, beta amyrin synthase; AAS, alpha amyrin synthase.

Cycloartenol synthase [(S)-2, 3-epoxysqualene mutase, EC 5.4.99.8] belongs to the 2–3 oxidosqualene cyclases (OCSs) gene family along with α-amyrin synthase (AAS), β-amyrin synthase (BAS), lupeol synthase (LS), and lanosterol synthase (LAS) [12–14]. Cycloartenol synthase (CAS) is one of the key enzymes involved in withanolide biosynthesis in *W*. *somnifera* (Fig 1). CAS is involved in cyclization of 2, 3-oxidosqualene to form cycloartenol which acts as the key precursor for the biosynthesis of phytosterols as well as withanolides through a series of desaturation, hydroxylation, epoxidations, cyclization, chain elongation, and glycosylation steps [15]. CAS from other plant species such as *Arabidopsis thaliana* [16] *Glycyrrhiza glabra* [17], *Pisum sativum* [18], *Rhizophora stylosa* [19], *Kandelia candel* [20] are also reported earlier. β-amyrin synthase (OCS) has been shown to be involved in the synthesis of glycyrrhizin and soyasaponin in Licorice [21]. Another OSC, shionone synthase is known to be associated with formation of a triterpene shionone in *Aster tataricus* [22]. Though some of the genes involved in withanolide biosynthesis have been cloned and characterized from *W*. *somnifera* [23–30]. Their further functional validation at plant level is still awaited. A close association of phytochemical profiling of medicinally important withanolides, organogenesis and expression of pathway genes has been established [31–32]. Transcriptomic data of withanolide biosynthetic pathway (leaf and root specific) were also reported which identified putative genes involved in withanolide biosynthesis [33–34].

Different genetic engineering methods, such as overexpression of transgenes, multiple expressions of transgenes, gene silencing, and transcription factors are powerful tools for the engineering of biosynthetic pathway [35]. In order to determine the gene function *via* transgenic approach two methods are very effective one of which involves loss of gene function i.e. RNAi mediated mechanism and other through gain of function or over-expression approaches. Both of these mechanisms together serve as an important tool to validate the function of a geneRNA-mediated gene silencing is initiated by the delivery of double-stranded RNA (dsRNA), which is processed into small interference RNA molecules (siRNA) of 21–25 bp by Dicer (DCR), which depends on ATP and RNaseIII. The siRNA is then incorporated into the RNAi-induced silencing complex (RISC), which in turn degrades the target mRNA [36]. As RNAi involves sequence-specific interaction between siRNA and mRNA, siRNAs can be tailored to silence a particular gene or even all the members of the gene family. -RNAi of specific target genes based on the stable expression of dsRNA has been successfully used in many economically important crop species [37–41]. Therefore, the study of functional role of candidate gene(s) dedicated to withanolide production via genetic transformation and functional genomics becomes an important issue in *Withania* research. In this study RNAi binary vector pGSA1131 expressing hairpin RNAs of *WsCAS* and pIGCAS (over-expression construct) were utilized to generate stable transgenic lines of *W*. *somnifera* to demonstrate the relation between *WsCAS* gene and withanolide production. Several transgenic silenced lines with reduced withanolide content were obtained as compared to control. In the same way transgenic *Withania* plants over-expressing *WsCAS* accumulated enhanced level of withanolides. Our study demonstrates that this transgenic approach could be efficiently used to improve and understand withanogenesis through functional genomics.

## Materials and Methods

### Plant Material

*Withania somnifera* Dunal plants were grown in glasshouse as well as in field in CIMAP-Experimental Farm at Lucknow, India, following standard agronomic practices. Seedlings were raised and planted in pots and field in the months of July and August and samples were harvested as per the experimental requirement from time to time and various experimental observations were recorded.

### Molecular cloning of *WsCAS*

Total RNA from *W*. *somnifera* leaf tissue was extracted by Trizol method and the first strand cDNA was synthesized using superscript II reverse transcriptase (Invitrogen), according to the manufacturer’s instructions. Sequences of primers were designed from the conserved domains identified in *CAS* gene reported from other plants. The PCR parameters were: initial denaturation at 94°C for 3 min, followed by 35 cycles at 94°C (20 sec), annealing at 56°C (40 sec), extension at 72°C (2 min) and final extension at 72°C for 7 min. The PCR products were electrophoresed on 1.2% agarose gel. The resolved amplicons were purified and cloned in vector pTZ57R/T (Fermentas, Life Sciences) and sequenced and deposited in NCBI database. On the basis of sequence information, a set of gene specific primers for 5’ and 3’ RACE were designed. The RACE amplified fragments were sequenced. On the basis of sequence information, a composite sequence of *WsCAS* encoding a full length cDNA (2.277 kb) was established. Another set of primers (*WsCAS*F0 and *WsCAS*R0) were designed to amplify complete strand of *WsCAS* and this full length was cloned in vector pJET1.2/blunt (Fermentas, Life Sciences) following standard protocols. Information related to all the primers used in this study was provided in S1 Table.

### Stress treatment

Seeds of *W*. *somnifera* were sown in field and one month old germinated seedlings were utilized for various stress treatments such as salicylic acid (SA), methyl jasmonate (MeJA), heat-shock, cold and wounding. For SA and MeJA treatment, seedlings were dipped in distilled water supplemented with 2 mMand 5 mM SA and 200 μM and 500 μM MeJA for 4 hrs. For heat shock treatment, seedlings were kept at 65°C for 1hr and for cold treatment seedling were kept at 4°C for 4 hr. Mechanical injury was caused by rubbing the leaves, and seedlings were dipped in distilled water for 4 hours.

### Silencing constructs preparation

The binary vector, pGSA (double-stranded RNAi vector) (ABRC Stock Number CD3-449), was used in this study to generate the *WsCAS* RNAi constructs. The pGSA vector contains an intron of the GUS gene of 372 bp to express CAS RNAi harpins. Three fragments of 261bp, 250bp and 323bp from *WsCAS* coding sequence from 5’, middle and 3’ prime regions respectively were amplified using the primers incorporating *Spe* I and *Asc* I in forward primer and *Bam* HI and S*wa* I in reverse primer as shown in bold letter in Table 1 and cloned in pJET vector and further sequenced. The amplification conditions were initial denaturation at 94°C for 3 min, followed by 35 cycles at 94°C (30 s), annealing at 55°C (40 s), extension at 72°C (1min) and final extension at 72°C for 7 min. The amplified fragment was initially digested with *Asc*I and *Swa*I and cloned in sense orientation into the binary pGSA1131 immediately after the CaMV 35S promoter to generate an intermediary vector. The same *WsCAS* fragment was then digested with *Spe*I and *Bam*HI and cloned in antisense orientation next to the GUS intron spacer region of the intermediary vector resulting in silencing construct WsRNAi 1, WsRNAi 2 and WsRNAi 3 respectively. The vector was sequenced to confirm the authenticity and correct orientation of the two cloned inserts.

**Table 1:** Primer Used in Study for Functional validation of *WsCAS*.

Primer Name	Primer Sequence	Amplicon length
WsCASF0	5’ GGA TCC ATG TGG AAG TTG AAG ATA GCA G 3’	2270 bp
WsCASR0	5’ GGA TCC TCA ATT AGC TTT GAG TAC ACG 3’	
WsCASSiF1	5’ GGACTAGTGGCGCGCC TATTCCACTCTACAAAATC 3’	293 bp
WsCASSiR1	5’ CGGGATCCATTTAAAT TCCTTCCCCAAGCAACCTC 3’	
WsCASSiF2	5’ GGACTAGTGGCGCGCC AAAATGAAGGGATACAATG 3’	282 bp
WsCASSiR2	5’CGGGATCCATTTAAAT GTCCCTCTGCAGTACAATC 3’	
WsCASSiF3	5’GGACTAGTGGCGCGCC AATTTTTTGTTGTCCAAAC 3’	355 bp
WsCASSiR3	5’CGGGATCCATTTAAATCGATATTCTCCCAATGCCC 3’	
nptII RP	5’ GCC AAC GCT ATG TCC TGA TAG C 3’	
WsCAS RTFP	5’- GGCTTGATTATTGCTCTA 3’	103 bp
WsCAS RTRP	5’- CACTGTTCTGATGGTTAT 3’	
Actin FP	5’- CTTTCTACAATGAGCTTCGTG 3	118 bp
Actin RP	5’- ATACAGTGAGAGAGGACAGCCTG 3’	

### Preparation of *WsCAS* over-expression construct

The full-length *WsCAS* sequence was amplified from leaf cDNA of *W*. *somnifera* with the forward *WsCAS*F0 and reverse *WsCAS*R0 primers incorporating *Bam*HI site at 5’ end as shown in bold letter in S1 Table. The amplified fragment was digested with *Bam*HI and cloned into pJET cloning vector (Fermentas). After sequence confirmation the desired insert was cloned at *Bam*HI site of binary vector pIG121Hm (AB 489142.1) resulting into overexpression vector pIGCAS.

### Generation of transgenic lines of *WsCAS* in *W*. *somnifera*

Node explants of *in vitro* grown seedling of *W*. *somnifera* were used for infection. Silencing constructs alongwith RNAi vector pGSA1131 and pIGCAS (overexpression construct) were used to transform *Withania* node explants. Stable transgenic *Withania* plants harboring these constructs were generated *via* optimized transformation protocol developed in our lab [42]. The pGSA1131 binary vector confers BASTA resistance in plants and hence transgenic silenced lines were selected by 50 μg L^-1^ BASTA. pIGCAS is harboring kanamycin as plant selectable marker gene thus overexpressing lines were selected on medium supplemented with 50 mg L^-1^ kanamycin.

### Molecular analysis of putative transformants

Plant genomic DNA was isolated using cetyl trimethylammonium bromide (CTAB) protocol with some modification from randomly selected silenced and overexpressing lines along with empty vector pGSA and untransformed plant. Genomic DNA from selected lines after BASTA/kanamycin selection was examined by PCR amplification for the presence of *bar* (phosphinothricin acetyl transferase gene conferring *BASTA* resistance) / *npt*II (neomycin phosphotransferase gene conferring kanamycin resistance) gene. The amplification conditions were initial denaturation at 94°C for 3 min, followed by 35 cycles at 94°C (30 s), annealing at 55°C (40 s), extension at 72°C (1:30 min) and final extension at 72°C for 7 min. Genomic DNA (15 μg) from randomly selected PCR-positive plantlets and non-transformed plant were digested with *Hind*III (silenced lines)/*Xba*I (over expressing lines), and the resulting fragments were separated by electrophoresis on 0.8% (w/v) agarose gel and immobilized on H^+^ nylon membrane (Sigma, USA) by capillary transfer method described by Sambrook and Russell [43]. Blots were hybridized with PCR-generated probes for *bar*/*npt*II gene (amplified as above), labeled with DIG-high prime, hybridization, washing, and detection were performed according to DIG-DNA labeling and detection kit according to manufacturer’s instruction (Roche Applied Science, Germany). The *bar*/*npt*II PCR product and genomic DNA from untransformed plants served as positive and negative controls, respectively.

### Quantitative Real-time PCR (qRT-PCR) analysis

Total RNA was isolated from shoot tissues using TRI reagent (Sigma, USA) and treated with RNase-free DNaseI (Fermentas). The first strand cDNA was synthesized from 5 μg of total RNA using RevertAid Premium first strand cDNA synthesis kit (Fermentas) according to manufacturer’s instructions (Fermentas). qRT-PCR was performed in transgenic silenced lines and over expressing *WsCAS* gene using the ABI real time DNA amplification system (Applied Biosystem, USA) under the following conditions: 95°C for 10 s; 40 cycles of 95°C for 5 s, 52°C for 20 s using *WsCAS* RT specific primers (Table 1). Melting curve analysis was included to verify specificity of the DNA amplification. The actin gene was used as reference gene. Ten-microliter reactions were set up for each sample in triplicate.

### HPLC analysis of withanolide content

Silenced lines, overexpressing lines of *WsCAS* and untransformed shoots were separated from the medium, frozen in liquid nitrogen, ground to a fine powder and were mixed with 1 ml 50% methanol and place for overnight with intermediate vigorous mixing. The extracted withanolides were dissolved in HPLC-grade methanol & used for HPLC analysis as reported earlier [44]. Total withanolide content (TWC) was defined as μg mg^-1^ of withanolides in fresh tissues used to assess the effects of *WsCAS* activity in withanogenesis.

## Results

### Cloning of *WsCAS*

Cycloartenol synthase gene occupies central position in the withanolide biosynthetic pathway (Fig 1). Different set of degenerate primers were designed on the basis of other known plant CAS gene sequences (S1 Table). Three partial *WsCAS* cDNAs were obtained using different pairs of degenerate primers and were designated as WSTS1 (GenBank: GU295060.1), WSTS2 (GenBank: GU295059.1) and WSTS3 (GenBank: GU295058.1). These partial WsCAS sequences showed very high sequence similarity with each other as well as exhibited close homology with *CAS* sequences in the NCBI database (S1 Fig). To make it full length gene 5’and 3’RACE was carried out from these partial fragments resulting in a single cDNA, *WsCAS*, of 2277 bps which was cloned using specific primer *WsCAS*F0 and *WsCAS*R0 (Table 1). The alignment of the *WsCAS* sequence with the sequences of reported *CAS*s revealed that *WsCAS* contained a putative open reading frame of 2277 bp encoding a polypeptide of 758 amino acid residues. The predicted polypeptide had a molecular weight of ~83 kDa. Deduced polypeptide showed sequence elements typical for CAS’s such as a DCTAE motif, QW motifs (S1 Fig). A phylogenetic tree was generated from the alignment of the deduced full-length amino acid sequences of twenty four CAS sequences reported from other plants. Despite being high level of homology among all CAS proteins, the phylogenetic analysis showed maximum similarity (92%) with *SlCAS* (S2 Fig).

### Modulation of *WsCAS* under different elicitor conditions

**Fig 2:**
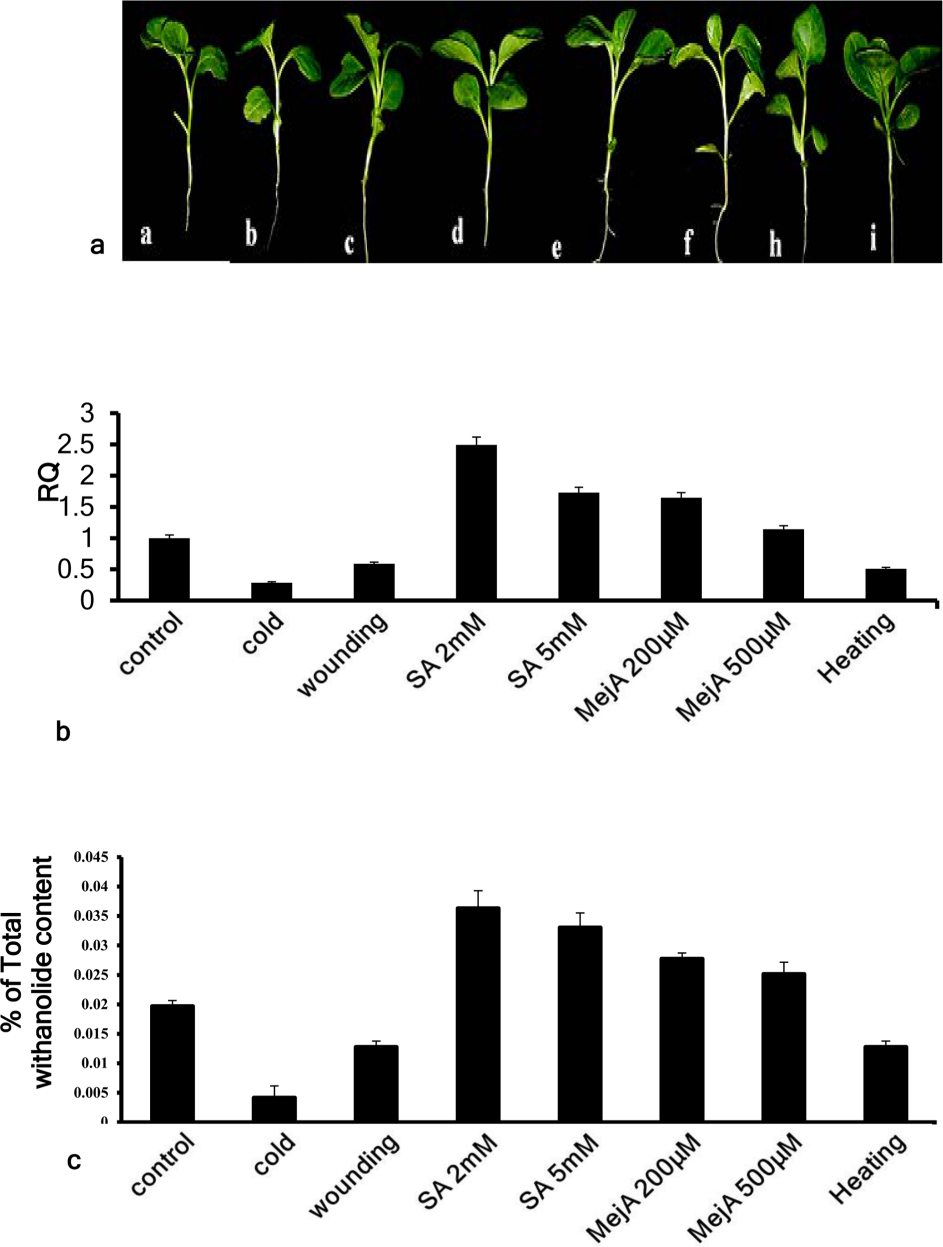
Profiling of *WsCAS* expression and withanolide in Withania seedlings under different elicitor conditions. (A) Seedling before and after treatment of cold, wounding, SA 2mM, SA 5mM, MeJ 200μM, MeJ 500μM and heating (a-i) respectively. (B) Real time-PCR analysis of *WsCAS*. (C) Quantitative withanolide profile.

Withania seedlings under different abiotic stress were shown in Fig 2A. SA 2 mM and 5 mM and MeJA 200 μM and 500 μM enhanced the *WsCAS* transcript expression levels upto 2.49, 1.72, 1.65 and 1.14 folds respectively while cold, heat and wounding lowered the *WsCAS* expression levels upto 0.25, 0.51and 0.59 fold respectively. To establish a link between *WsCAS* transcript accumulation and withanolide levels under the above conditions, phytochemical analysis was also carried out. The overall content of withanolide was higher in case of SA and MeJA treatments at both the concentrations while lower in cold, heat and wounding in comparison with control respectively. Percentage of total withanolide at SA 2 mM and 5 mM was found increased upto 2.0 and 1.75 folds, respectively as compared to control. In case of MeJA treatment withanolide content was increased upto 1.42 fold at 200 μM and upto 1.31 fold at 500 μM while decreased by 0.2 fold under cold stress conditionand 0.63 folds under both, heat and wounding treatments. Our results revealed a direct correlation between the *WsCAS* expression and accumulation of withanolide in treated seedlings under elicitor (SA and MeJ) treatment and heat, cold and wounding stress conditions (Fig 2).

### Designing of silencing constructs

**Fig 3:**
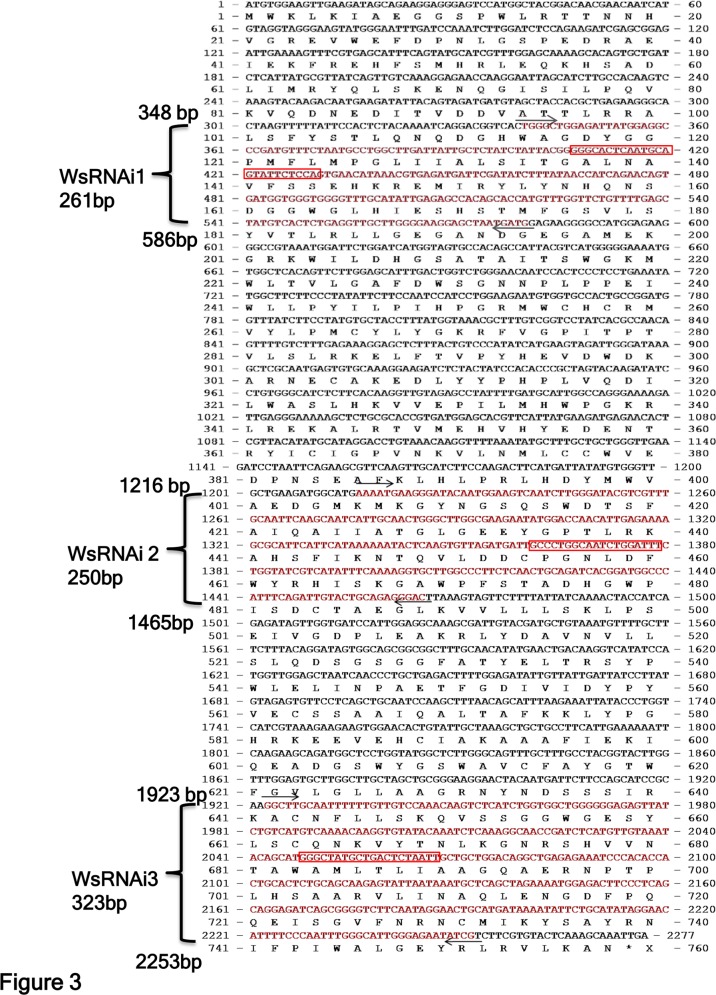
Full length *WsCAS* gene with three selected silencing sites for silencing constructs preparation in *Withania somnifera*. 19 bp silencing sites were enclosed in red colored boxes. Arrows indicate the region of start of amplification of particular fragment incorporating silencing sites.

**Fig 4:**
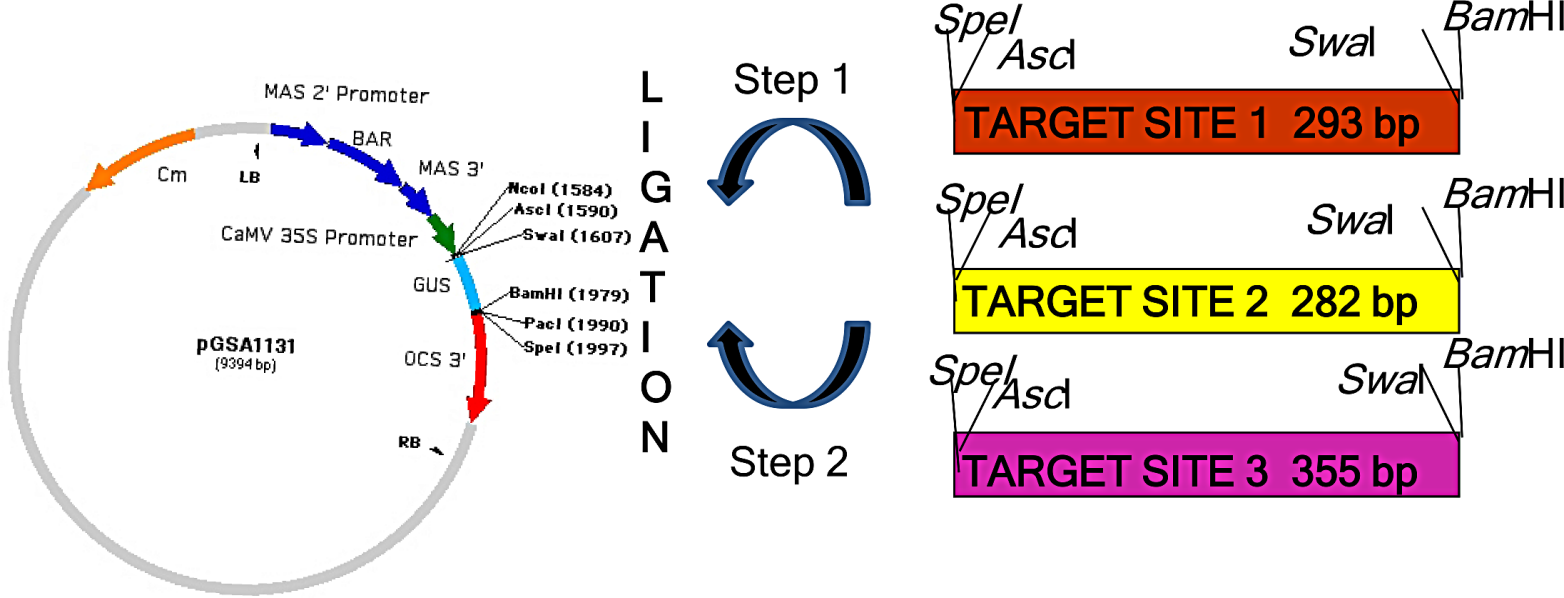
Sequence analysis of *WsCAS*. Alignment of predicted amino acid sequence of newly identified *WsCAS* cDNA with the cycloartenol synthases reported in the gene bank from various plants such as *Lotus japonicus* (AB181246), *Arabidopsis thaliana* (NM_126681), *Solanum lycopersicum* (EU449280), *Panax notoginseng* (EU342419) and *Bupleurum kaoi* (AY514456) showing conserved domains. The predominant DCTAE motif (highlighted in yellow), five QW motifs (underlined) and three conserved amino acids- Tyr410, His477, Ile481 (depicted by symbol♦) are appropriately shown.

**Fig 5:**
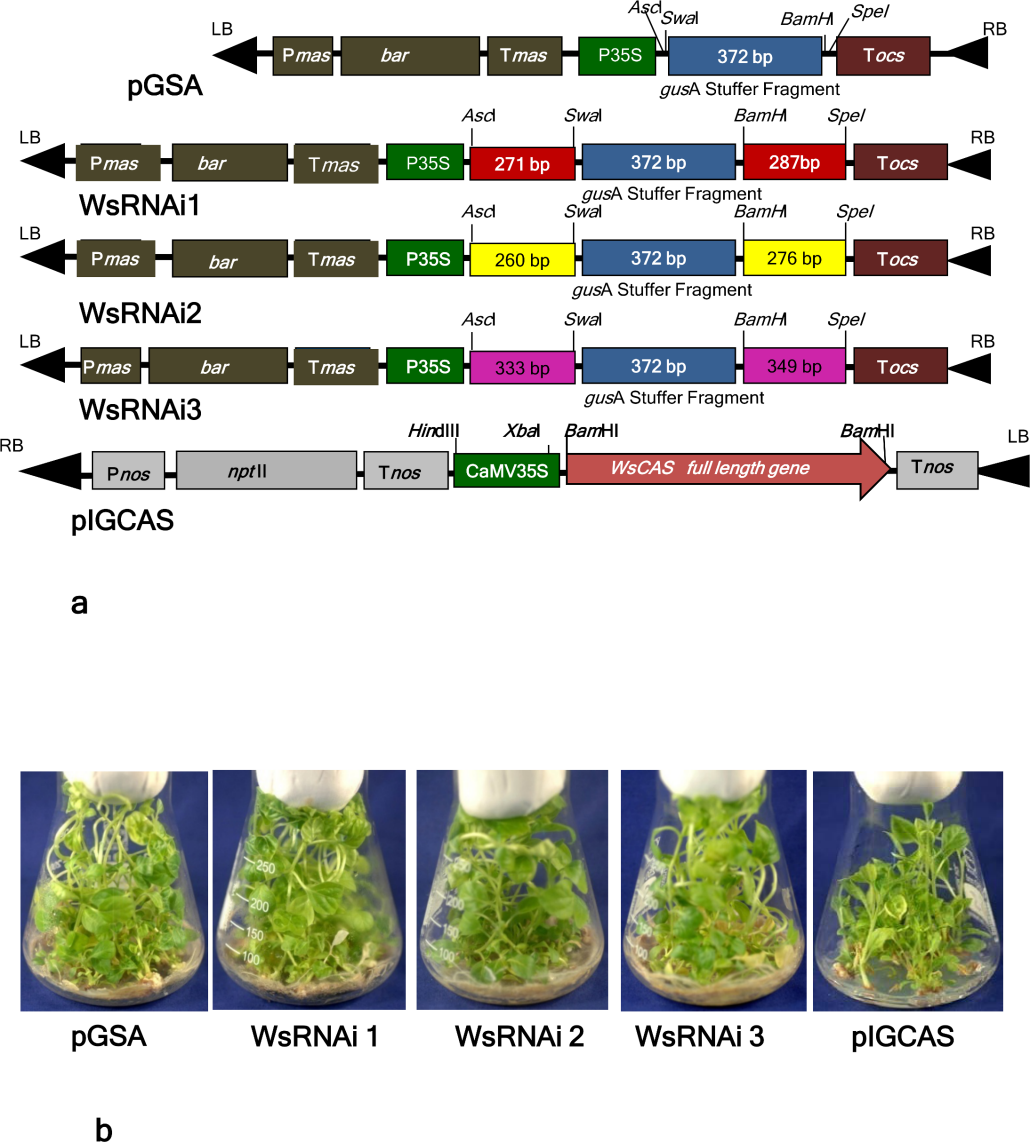
(A) T-DNA region of constructs. BAR, gene encoding resistance to the herbicide BASTA; OCS 3', poly adenylation signal sequence; MAS 3', poly adenylation signal sequence; MAS 2', plant promoter; CaMV 35S’ Viral promoter; LB, left border; RB, right border; GUS, 360 base pair fragment from the GUS gene; P promoter; T terminator; *nos* nopaline synthase; *npt*II neomycin phosphotransferase gene. (B) Generation of *W*. *somnifera* transgenic lines.

Since higher silencing efficiencies were desired, the full length *WsCAS* cDNA sequence was analyzed for strong putative silencing sites. Three sites were selected and tested in small inverted repeats, first inverted repeat (IRs) comprising 261 bp from the 5’ region, second one of 250 bp from middle region and the third 323 bp from 3’ region respectively (Fig 3). For silencing construct preparation a binary RNAi vector pGSA1131 was used. The three selected sites were amplified using Forward primer incorporating *Spe*I and *Asc*I sites and reverse primer having *Bam*H1 and *Swa*I restriction enzyme sites (shown in bold letters in Table 1). The amplified products were cloned in cloning vector pJET (Fermentas) and sequenced. The cloned plasmids were characterized via colony PCR and restriction digestion. These cloned *WsCAS* fragments digested with *Asc*I and *Swa*I of 271bp, 260 bp and 333 bp respectively from 5’, middle and 3’ region were cloned in sense orientation into the binary double-stranded RNA vector pGSA1131 immediately after the CaMV 35S promoter to generate an intermediary vector. The same *WsCAS* fragment was then digested with *Spe*I and *Bam*HI of 287 bp, 276 bp and 349 bp respectively from 5’, middle and 3’ region and cloned in antisense orientation next to the GUS intron spacer region of the intermediary vector resulting in RNAi silencing constructs WsRNAi 1, WsRNAi 2 and WsRNAi 3. Transcription of these construct would be expected to give a hairpin RNAs (Fig 4). The total cassette including sense and antisense fragments along with GUS spacer region were checked by cutting it with *Asc*I restriction enzyme and the total length of the inserted fragment were 923, 901, and 1047 bp, as expected respectively from 5’, middle and 3’ region of *WsCAS*. DNA sequencing confirmed that these *WsCAS* RNAi vectors were constructed successfully. The T-DNA regions of these silencing vectors were shown in Fig 5A.

### Preparation of *WsCAS* over-expression constructs

The full-length *WsCAS* sequence was amplified from cDNA of Withania with the forward *WsCAS*F0 and reverse *WsCASR*0 primer incorporating *Bam*HI site at 5’ end (Table 1). The amplified fragment of *WsCAS* full length (2277 bp) was digested with *Bam*HI and cloned at *Bam*HI site of binary vector pIG121Hm (AB 489142.1) resulting into over-expression vector pIGCAS. The T-DNA region of pIGCAS vector was also shown in Fig 5A.

### Generation of transgenic plants harboring silencing and over-expression constructs

These silencing constructs along with vector control pGSA and over-expression construct were finally transformed in *Agrobacterium* strain GV3101 and after characterization used for plant transformation experiments. Stable transgenic Withania plants harboring these constructs were generated *via* developed transformation protocol using binary vector pIG121Hm from node explants [42]. Briefly, explants after 10–12 days of infection, transferred to selection media having BASTA 50 μg l^-1^ or kanamycin 50 mg l^-1^ for three consecutive selection cycles. All transformants were morphologically similar to untransformed one. The only difference is that silencing lines generated after BASTA selection, while in case of over expressing lines selection was on kanamycin (Fig 5B).

### PCR and southern blot analysis

**Fig 6:**
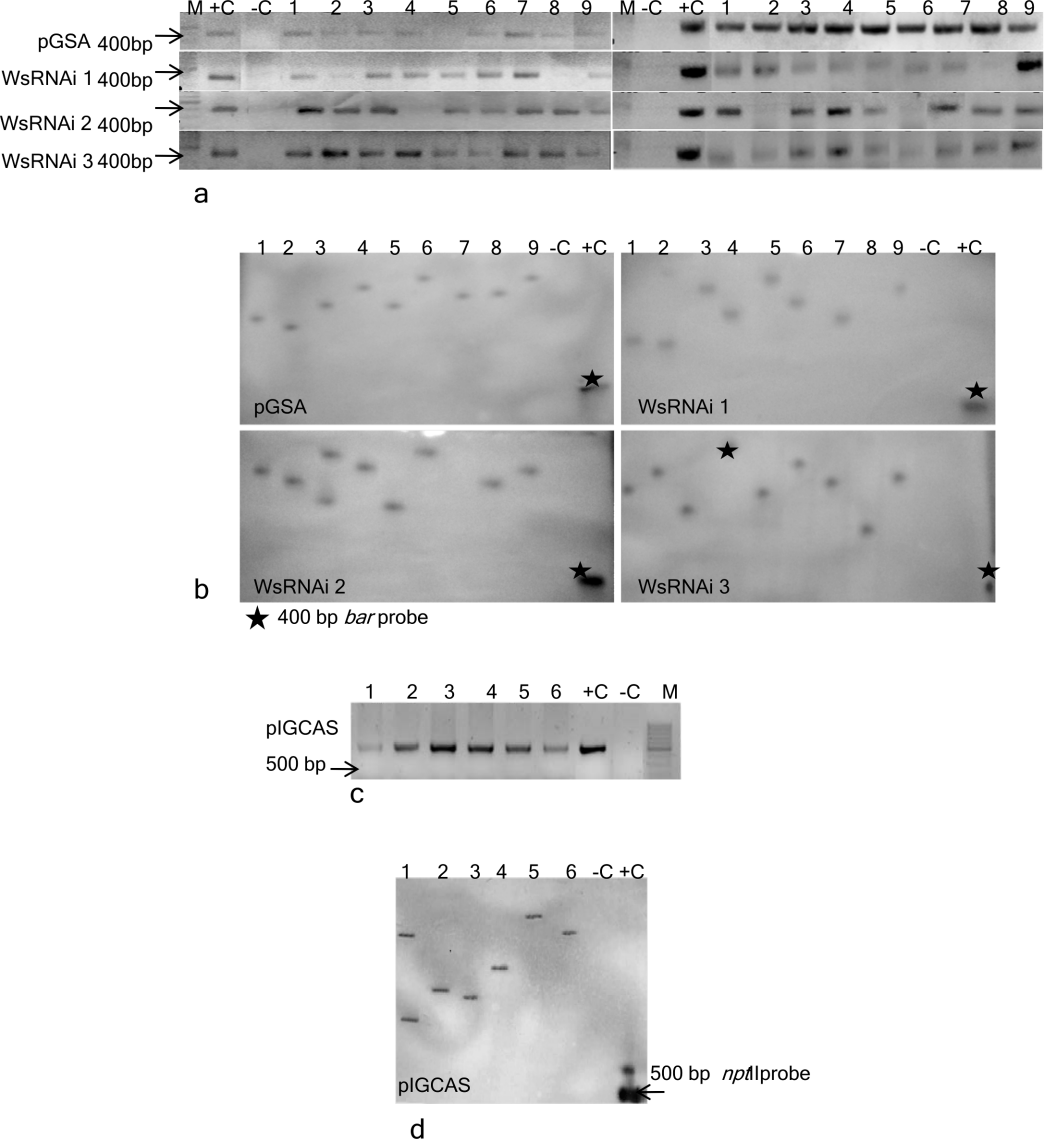
Molecular characterization of Withania silenced lines and overexpressing lines of *WsCAS gene*. (A) PCR amplification with *bar* primers showing amplicon of ≈400 bp. Lane M 100 bp ladder; lane–C untransformed shoot; lane +C plasmid DNA; lane 1–9, *in vitro* transformed shoots; (B) Southern blot analysis of transgenic silenced lines of *Withania* with *bar* PCR probe. (C) PCR amplification of pIGCAS transformants with *npt*II primers showing amplicon of 500bp. Lane- 1 to 6 transformed lines. (D) Southern blot analysis of overexpresing lines.

The plants after three consecutive cycles of BASTA/kanamycin selection were used for molecular analysis. The PCR amplifications with genomic DNA from silenced lines as well as vector pGSA transformed lines showed 418 bp amplicon for *bar gene* (phosphinothricin acetyl transferase gene conferring *BASTA* resistance) on agarose gel (1.0%, w/v) electrophoresis of the amplification reaction mixture (Fig 6A). In the same way PCR analysis of over expressing lines of randomly selected putative T_0_ transgenic plant showed desired amplicon of *npt*II of 500 bp (Fig 6C). No amplification was detected in the untransformed (control) plants. To confirm the integration and copy number of transgene in *W*. *somnifera* plantlets, Southern blot hybridization was performed on total genomic DNA isolated from BASTA resistant (silenced lines along with vector pGSA transformed line) and kanamycin resistant (over expressing lines) and untransformed (control) plantlets. *bar* and *npt*II PCR products were loaded as positive control. Genomic DNA was digested with *Hind*III, which has unique site within the T-DNA region of silencing constructs and with *Xba*I in case of overexpressing lines. The blot was probed with the PCR-amplified product of *bar* (418bp) *and npt*II (500bp) gene there by generating a unique fragment for each integrated copy. Genomic DNA of randomly selected silenced lines alongwith vector pGSA transformed lines as well as overexpressing lines showed hybridization signal with digoxigenin-labelled *bar* and *npt*II gene (PCR product 418 bp & 500 bp respectively) probe (Fig 6B and 6D). No hybridization signal was observed in the control plant. The position of hybridization signal varies among different regenerated transgenic plantlets indicating these transgenic plants arose as independent transformation events.

### Expression analysis *WsCAS* transcript level in transformants

**Fig 7:**
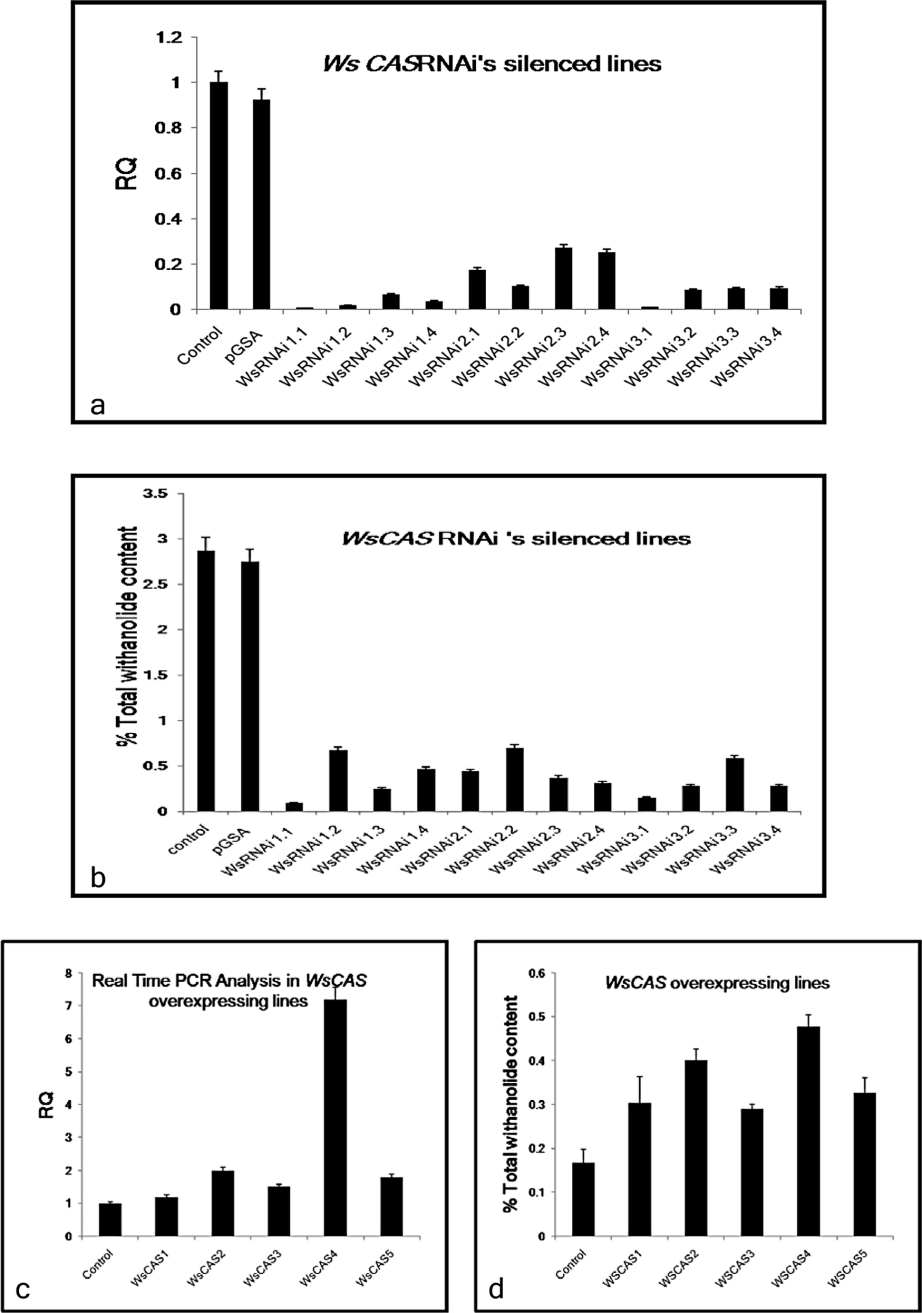
*WsCAS* transcript expression analysis and withanolide accumulation in Withania silencing and overexpressing lines. (A) RealTime PCR analysis of *WsCAS* silenced lines. (B) Withanolide content in silenced lines. (C) Real-Time PCR analysis of *WsCAS* overexpressing lines. (D) Withanolide content in *WsCAS* overexpressing lines.

Quantitative real time-PCR was carried out to check the transcription level of *WsCAS* in the transgenic silenced lines generated with different RNAi constructs and in over-expressing lines as well. The results showed that the transcription level of *WsCAS* was reduced by approximately 80–97% as compared to the control from 5’, middle and 3’ region (Fig 7A). WsRNAi 1 showed the maximum suppression of mRNA levels upto 96.7% followed by WsRNAi 3 (92.8%) and WsRNAi 2 (79.9%) on average basis. As expected, the transgenic *WsCAS* overexpressing lines showed enhancement of 1.2 to 7 fold of *WsCAS* transcript level, as compared to untransformed control plants (Fig 7C).

### Metabolite Analysis

HPLC analysis was performed to estimate the withanolides in stable transgenic silenced lines as per protocol developed and used in our earlier reports [1–6]. The silenced lines had altered withanolide profiles, as revealed by HPLC (S3 Fig). These results indicate that there was 33–91% reduction in withanolide content in silenced lines (Fig 7B).Variation was seen in withanolide content among three different target sites i.e. WsRNAi 1, WsRNAi 2 and WsRNAi 3 and also within the transgenic lines of these sites. Withanolide content reduced in WsRNAi 1 lines from 33–91% followed by WsRNAi 2(30–69%), WsRNAi 3 (42–91%). Although variation in withanolide content was seen among different independent lines transformed with same construct but generalization of the result showed that among three sites withanolide content was lowest in WsRNAi 1 followed by WsRNAi 3 and WsRNAi 2. In case of over-expressing lines withanolide content was increased to the extent of 1.06 to 1.66 fold (Fig 7D).

## Discussion

Oxidosqualene cyclases (OSC’s) serve as key enzymes in secondary metabolite synthesis by driving the metabolic pool of isoprenoids and converting it to a range ofhigher terpenoid- secondary metabolites *via* cyclization of 2, 3 oxidosqualene [12–14]. Cycloartenol synthases (CAS), an important OCS have been cloned from other plant species also such as *Glycyrrhiza glabra*, *Oryza sativa* licorice, pea etc. and its involvement in terpenoid biosynthesis has also been studied [16–20]. It has been established that under stress related conditions elicitors help in increasing the concentration of metabolites and provides resistance against various biotic and abiotic stresses. However when a fungal elicitor protein, cryptogein encoding gene was co-transformed in hairy roots of *W*. *somnifera*, withanolide content and expression of pathway genes such as HMGR, FPPS were down-regulated. The co-transformation resulted in shift of metabolic pool from withasteroid formation to phenylpropanoid formation [45]. Secondary metabolites also have defensive role so an attempt was made to understand the linkage between elicitor treatment and expression of CAS gene. Real time PCR results showed that *WsCAS* was up regulated by SA and MeJA (signalling molecules) while down regulated by cold, heat and wounding stress.Expression of *WsDXS* and *WsDXR* in response to exogenous application of SA, MeJA, and wound treatments were also studied earlier showing their involvement in early steps of withanolide biosynthesis [24]. A similar result was observed in case of *WsFPPS* gene in response to different elicitors [23]. Modulations in the expression of *WsHMGR* in response to exogenous applications of SA and MJ as well as to mechanical injury was studied earlier [Akhtar et al. 2013 26]. Our findings suggest that expression level of *WsCAS* was modulated in response to heat cold, wound, MeJA, and SA treatments in condition specific manner. HPLC analysis showed the relative change in withanolide content also (Fig 2). These finding supports that withanolide biosynthesis is tightly regulated by *WsCAS* in *W*. *somnifera*.

Despite being indicated several times CAS as an important enzyme in withanolide biosynthetic pathway by various researchers, hardly attempts were made to validate the function of the gene *in planta*. Transgenic approaches provide a powerful tool for metabolic engineering and gene function studies in Withania plants. Based on the improved transformation system and development of frequent changes and progression in the field of RNAi, we carried out the study on the gene function investigation by RNAi technology. RNAi mediated gene silencing studies was an effective measure to carry out gene function investigation and further improvement in crop plant species [46] In tobacco 9.1–96.7% reduction in nicotine levels was observed on silencing of putrescine N-methyltransferase (PMT) gene [47]. Similarly transgenic RNAi silenced lines for caffeine synthase showed reduction in levels of caffeine and theobromine in *Camellia sinensis* [48]. When an OSC, dammarenediol synthase (DDS) which is involved in ginsenoside synthesis was silenced by RNAi in *Panax ginseng*, significant reduction in transcript level and subsequently ginsenoside production was observed [49]. In present study we constructed three silencing vectors for *WsCAS* gene spanning various regions of the gene (WsRNAi 1, WsRNAi 2 and WsRNAi 3) of full length *WsCAS* (Fig 5A). Most importantly the entire cassette including sense and antisense fragments along with GUS spacer region were transferred to any other plant transformation vector by cutting single enzyme *Asc* I (Fig 5A).

Silencing of related genes is highly efficient after infiltrating leaves with *Agrobacterium* transformed with the RNAi vector containing two inverted-repeat sequences separated by an intron in many earlier reports [50]. RNAi-mediated silencing of the flavanone 3-hydroxylase gene in transiently transformed strawberry fruits was studied and found downregulation of F3H gene by approximately 70% in the agroinfiltrated fruits compared with the control and also showed reduced flavonol and anthocyanin content [37]. These transformation methods gave first clue for understanding of both biosynthetic and regulatory genes and provide potential means to improve the specificity and effectiveness of particular metabolite biosynthetic pathway. But further such metabolic engineering efforts augment well after generation of stable transgenic lines within short time for the manipulation of metabolic flux towards efficient biosynthesis of desired secondary metabolites. RNA interference of squalene epoxidase 1 gene in transgenic *Panax ginseng* completely suppressed *PgSQE*1 transcription. Concomitantly, the interference of *PgSQE1* resulted in 50% reduction of ginsenoside production [39]. As silencing of a gene helps in determination of gene function another way for characterization of a gene is its over-expression. It offers the advantage that apart from functional validation of a gene it helps in increased production of metabolites. Over-expression involves increasing the level of genes either by transcriptional enhancers mediated random activation of endogenous genes or by the expression of individual transgenes by transformation [51]. Keeping this in view *WsCAS* gene was over-expressed using pIG121 as binary vector. This gene was selected for testing RNAi in *Withania* because of catalyzing penultimate step of withanolide biosynthesis. Large numbers of independent transformants were produced for each construct. During the course of experiment no significant visible changes were observed in growth and development pattern of transgenic lines in comparison to untransformed lines (Fig 5B).

Molecular analysis of putative T_0_ transformants was carried out by PCR amplification of the *bar/npt*II fragment for the silenced transgenic lines and over-expressed lines respectively. Amplification of *bar* was observed in all silenced transgenic lines along with pGSA vector and over-expressing lines showed positive amplification for *npt*II gene. The Southern blots were performed with *Hind*III (in case of silenced lines) or *Xba*I (overexpressing lines) digested genomic DNA from the shoot of T_0_ transgenic plants, and probed with a DIG-labeled DNA probe of partial PCR amplified fragment of *bar/npt*II gene. The results showed the *bar/npt*II sequence was present in all T_0_ transgenic plants analyzed, and mostly each line contained single integrated loci of the *bar/npt*II gene (Fig 6). Southern blot also showed that the integration of transgene is a random event and not site specific and hence may result in different pattern of enzymatic activity. *Agrobacterium* mediated transformation generally results in integration of low copy number of T-DNA. Single copy of transgene provides stability to transgenic lines.

The results showed that the transcription level of *WsCAS* was reduced by approximately 80–97% as compared to the control from different target regions of the gene. WsRNAi 1 showed the maximum suppression of mRNA levels followed by WsRNAi 3 and WsRNAi 2 on average basis. The 5’ IR construct (WsRNAi 1) gave a higher silencing efficiency in range from 93.3 to 98.9% than the middle (WsRNAi 2) and 3’ IR (WsRNAi 3) construct. Withanolide content from the transformed plants was analyzed by HPLC analysis. Several silenced lines with low levels of withanolide content were obtained, in which, withanolide content decreased considerably showing the down regulation mediated by RNAi of *WsCAS*. The results demonstrated that the withanolide content of transformants differed significantly in silenced lines with varying levels of individual withanolide as compared to the untransformed one. Manipulation of saponin biosynthesis by RNA interference-mediated silencing of b -amyrin synthase gene in soybean exhibited a stable reduction in seed saponin content, correlated with the b -amyrin synthase mRNA depletion [41]. In tomato silencing of lycopene β and ε-cyclase genes was resulted in reduction of gene expression upto 8.95% and 13.16%, respectively [52]. Similarly, RNAi-mediated suppression of p-coumaroyl-CoA 3’-hydroxylase in hybrid poplar resulted in down-regulation of C3’H in a number of transformed lines, which generally correlated very well with reduced total cell wall lignin content [53]. From this we concluded that the down-regulation of *WsCAS* gene expression was due to the formation of the *WsCAS* RNA hairpin *in vivo*, which resulted in a decrease in *WsCAS* enzyme activity, ultimately resulting in a reduction in the biosynthesis of withanolides (Fig 7). In the same way *WsCAS* overexpressing lines showed enhancement of upto 7 fold of *WsCAS* transcript levels. An increase in withanolide content was maximally upto 1.66 fold was noticeable in over-expressed transformants (Fig 7). In *Catharanthus roseus* over-expression of a regulatory gene (ORCA3) and a structural gene (G10H) involved in MIA pathway increased the accumulation of monoterpenoid indole alkaloids [54]. Overexpression of CrtR-b2(carotene beta hydroxylase 2) in transgenic tomato plants resulted in elevated xanthophyll content [55].

Therefore, inverted repeat silencing construct under the control of the constitutive promoter were able to effectively down-regulate the pathway gene expression in *Withania*. However variation in direct linkage between CAS transcript level and relative withanolide content was observed in transgenic lines. The reason for that withanolide is not the immediate product of cycloartenol synthase. The withanolide biosynthesis involves various post-transcriptional and post- translation modification steps [3]. Difference in withanolides arises due to modification in carbocyclic skeleton or the side chain. The plant exhibits enzymatic machinery which can oxidize all the carbon atoms in steroid nucleus [9]_._ Studies on biosynthetic pathways and metabolic engineering already showed that the fluxes through the pathways are controlled not only by gene expression levels, but also by post-translational regulation of enzyme activity and enzyme and metabolite compartmentalization and transport [56]. Also the variation within these lines may be attributed to variation in expression levels of transgenes.

In conclusion, the present study was an attempt to generate transgenic lines containing a construct which impairs the normal functioning of a key pathway enzyme cycloartenol synthase (CAS) thereby suppressing withanolide biosynthesis in *W*. *somnifera* and also generating over-expressed lines with elevated levels of withanolides. Strategies utilized in both the experiments were different but it complemented with each other in the sense that both helped in determining the function of the *WsCAS* gene. The observed decrease in *WsCAS* transcription level in *Withania* silenced lines very likely resulted in a reduction in *WsCAS* enzyme activity *in vivo*, which in turn responsible for reduction in the biosynthesis of withanolides by regulationg a key position in the biosynthetic pathway by *WsCAS*.

## Supporting Information

S1 Fig Sequence analysis of *WsCAS*. (TIF)

S2 Fig Phylogenetic tree of CAS. (TIF)

S3 Fig HPLC chromatograms of withanolide analysis in Withania transgenic lines. (a) silencing lines. (b) over expressing lines.
(TIF)

S1 Table Primers used for molecular cloning of cycloartenol synthase from *Withania somnifera*. (DOC)
