# Supplementary figures and images for: RNAi and Homologous Over-Expression Based Functional Approaches Reveal Triterpenoid Synthase Gene-Cycloartenol Synthase Is Involved in Downstream Withanolide Biosynthesis in Withania somnifera
Source: PLoS One. 2016 Feb 26;11(2):e0149691. doi: 10.1371/journal.pone.0149691 (PMC4769023; doi:10.1371/journal.pone.0149691)

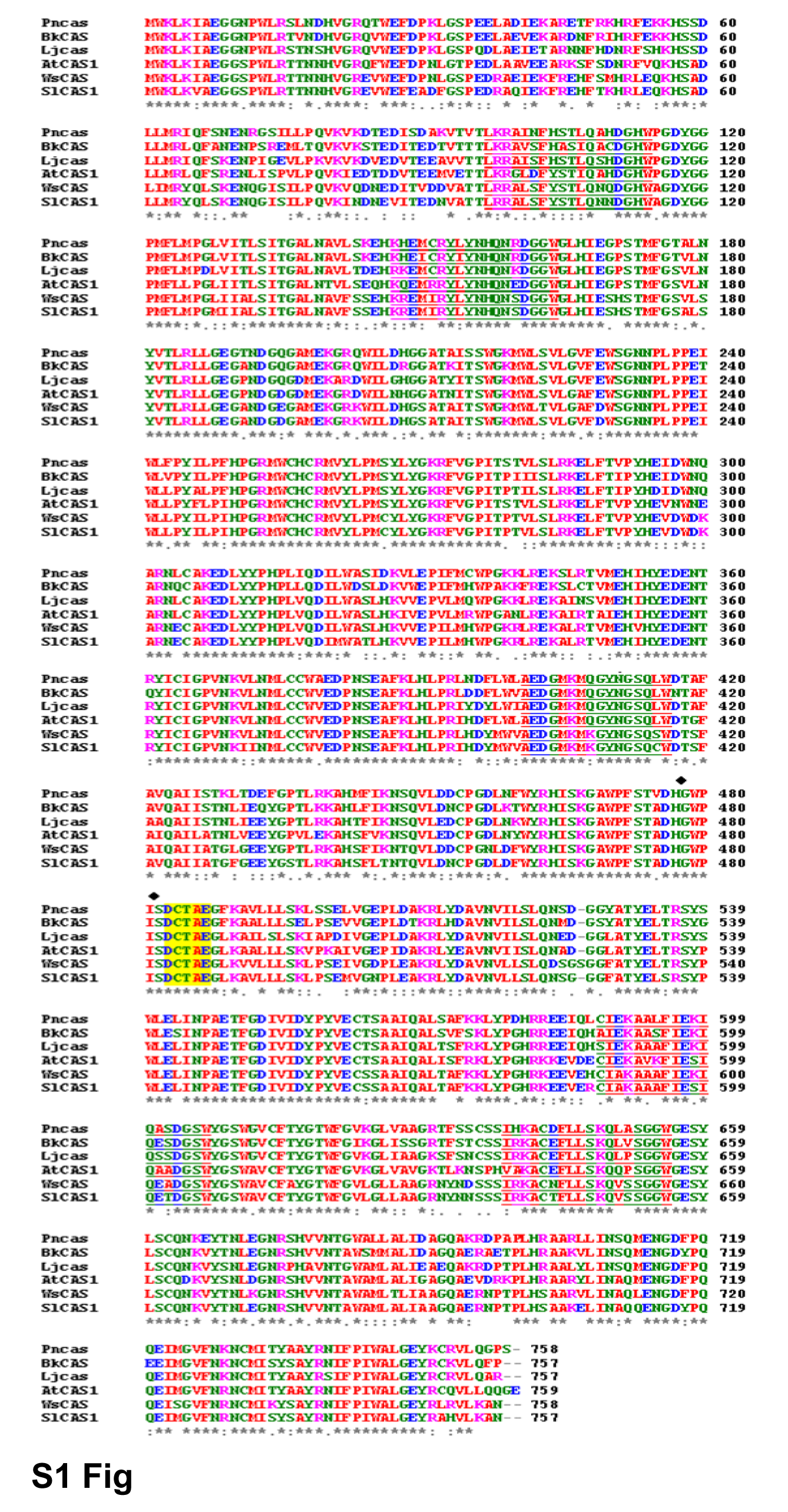

Supplement: S1 Fig — (TIF) [file pone.0149691.s001.tif]

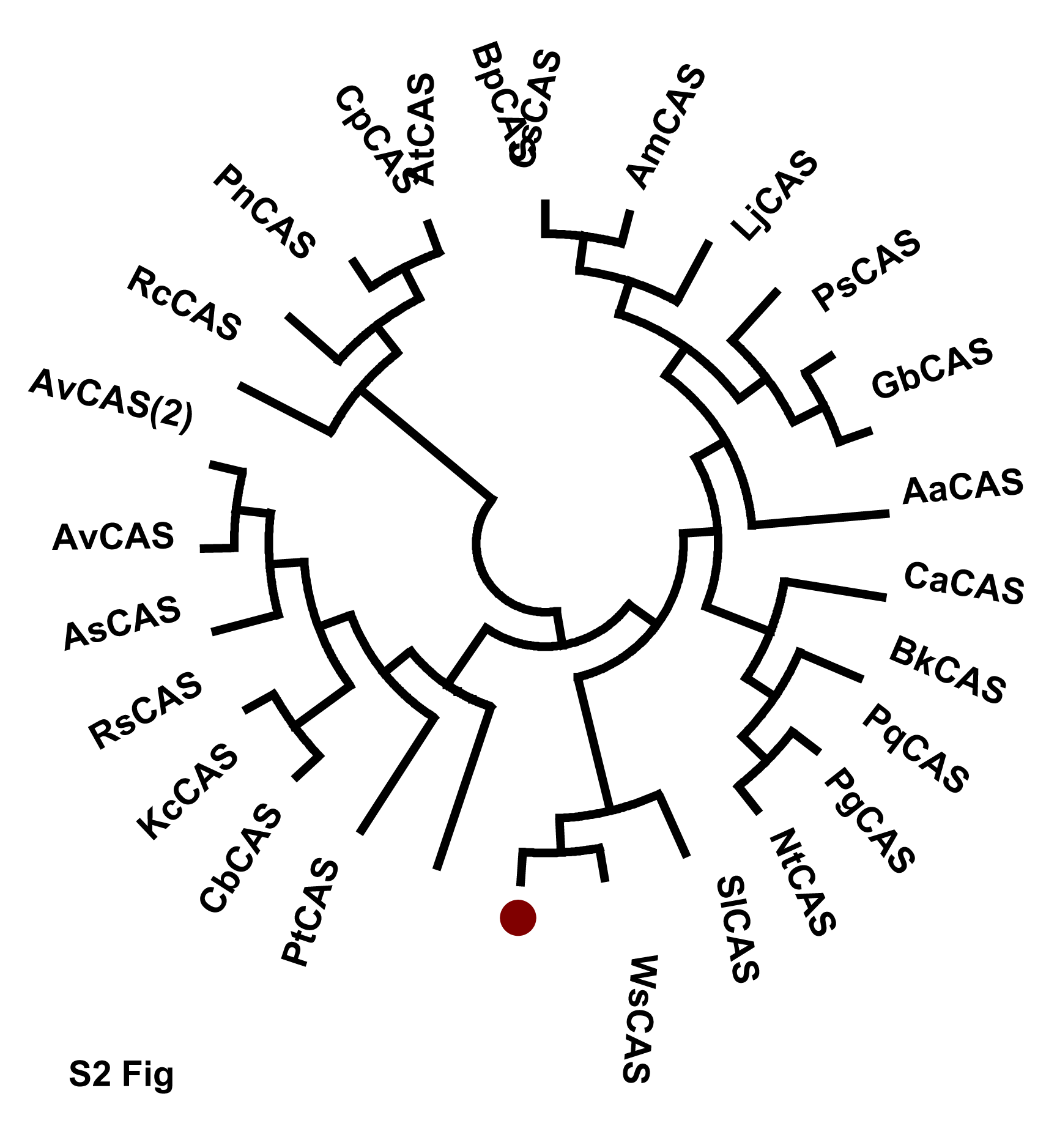

Supplement: S2 Fig — (TIF) [file pone.0149691.s002.tif]

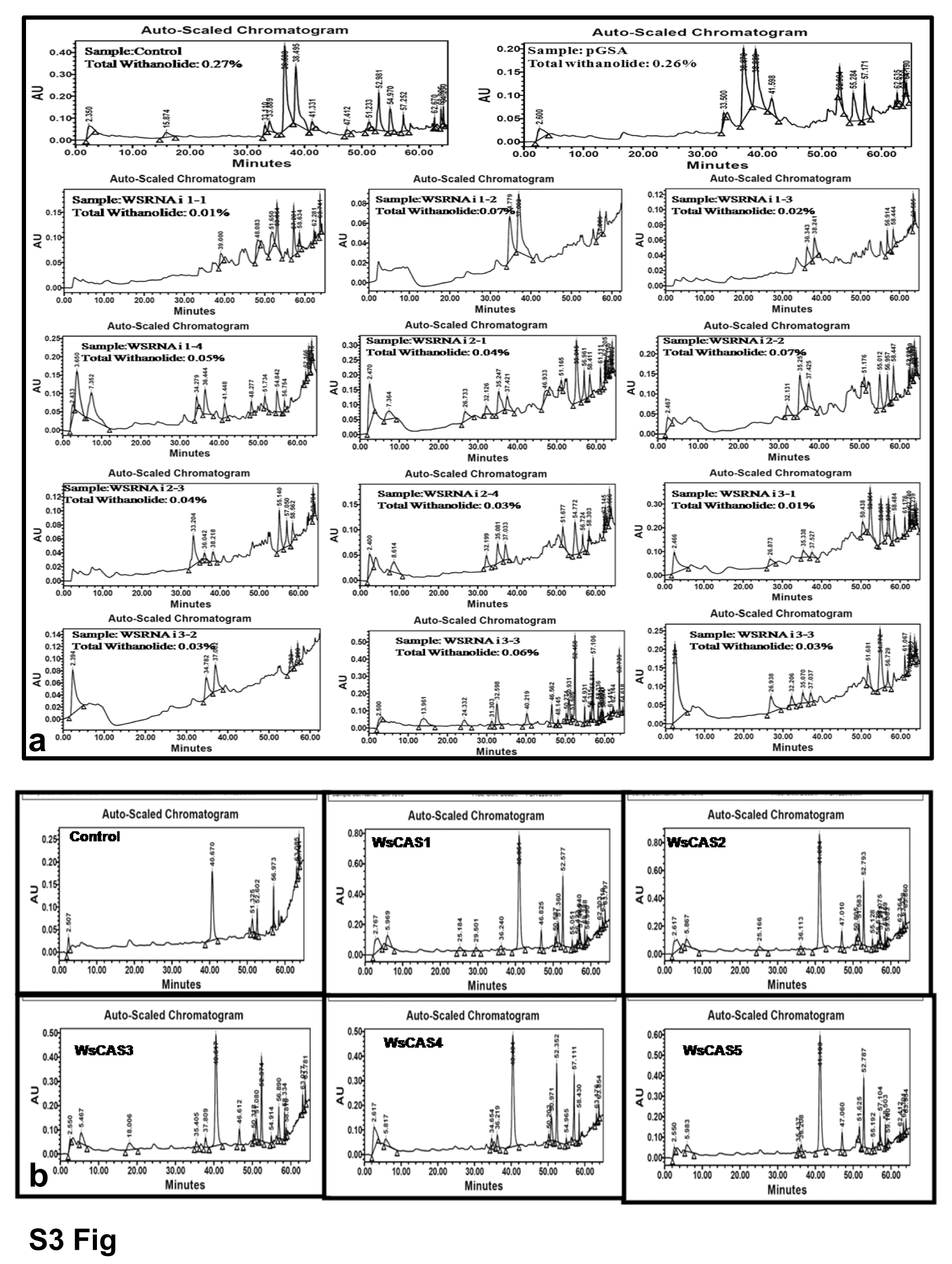

Supplement: S3 Fig — (a) silencing lines. (b) over expressing lines. (TIF) [file pone.0149691.s003.tif]
