# Supplementary material for: RNAi and Homologous Over-Expression Based Functional Approaches Reveal Triterpenoid Synthase Gene-Cycloartenol Synthase Is Involved in Downstream Withanolide Biosynthesis in Withania somnifera
Source: PLoS One. 2016 Feb 26;11(2):e0149691. doi: 10.1371/journal.pone.0149691 (PMC4769023; doi:10.1371/journal.pone.0149691)
Supplement: S1 Table — (DOC) [file pone.0149691.s004.doc]

**S1 Table.** **Primers used for molecular cloning of cycloartenol synthase from *Withania somnifera.***

| Primer Name | Primer Sequence | Use |
| --- | --- | --- |
| casF-1 | 5’ GAY GGN GGN TGG GGN YTN CA 3’ | Cloning of partial CAS fragment |
| casR-1 | 5’ NGT RTY YTC RTC YTC RTA RTG 3’ | Cloning of partial CAS fragment |
| casF-2 | 5’ TGY GCN AAR GAR GAY YTN TAY TA 3’ | Cloning of partial CAS fragment |
| casR-2 | 5’ RCA RTC NSW DAT NGG CCA NCC RTC 3’ | Cloning of partial CAS fragment |
| casF-3 | 5’ ATH GGN CCN GTN RAY AAR G 3’ | Cloning of partial CAS fragment |
| casR-3 | 5’ DAT RTC NCC SAA NGT KTC NGC NGG 3’ | Cloning of partial CAS fragment |
| casF-A | 5’ ATG TGG AAR YTN AAR GTN GC 3’ | Cloning of partial CAS fragment |
| casR-A | (5’ RTG NAR NCC CCA NCC NCC RTC 3’ | Cloning of partial CAS fragment |
| casNF-1 | 5’ TGG GGA AGG AGC TAA TGA TG 3’ | Cloning of partial CAS fragment |
| casNR-1 | 5’ TCC AAT GGA TTA CCG ACC AT 3’ | Cloning of partial CAS fragment |
| casNF-2 | 5’ TGC CAC AGC CAT TAC ATC AT 3’ | Cloning of partial CAS fragment |
| casNR-2 | 5’ CCA ATG GAT TAC CGA CCA TC 3’ | Cloning of partial CAS fragment |
| casNF-3 | 5’ TGC CAC AGC CAT TAC ATC AT 3’ | Cloning of partial CAS fragment |
| casNR-3 | 5’ TCC AAT GGA TTA CCG ACC AT 3’ | Cloning of partial CAS fragment |
| casNF-4 | 5’ TAG CAG AAG GAG GGA GTC CA 3’ | Cloning of partial CAS fragment |
| casNR-4 | 5’ GAA CCA AAC ATG GTG CTG TG 3’ | Cloning of partial CAS fragment |
| casNF-5 | 5’ TAG CAG AAG GAG GGA GTC CA 3’ | Cloning of partial CAS fragment |
| casNR-5 | 5’ TGC TGT GGC TCT CAA TAT GC 3’ | Cloning of partial CAS fragment |
| WsCAS-R1 | 5’ CGCATAATGAGATCAGCACTGTGC 3’ | 3’ RACE |
| WsCAS-R2 | 5’ CTGTGCTTTTGCTCCAAACGATGC 3’ | 3’ RACE |
| WsCAS-R3 | 5’ TCCGCTCGATCTTCTGGAGATCC 3’ | 3’ RACE |
| WsCAS-F1 | 5’ TGG AGG CCC GAT GTT TCT AAT GCC TGG C 3’ | 5’ RACE |
| WsCAS-F2 | 5’ TGC TCT ATC TAT TAC GGG GGC ACT C 3’ | 5’ RACE |
| WsCAS-F3 | 5’ CCA TCA GAA CAG TGA CGG 3’ | 5’ RACE |
| *WsCAS*F0 | 5’ **GGA TCC** *ATG* TGG AAG TTG AAG ATA GCA G 3’ | Full length cloning of *WsCAS* |
| *WsCAS*R0 | 5’ **GGA TCC** *TCA* ATT AGC TTT GAG TAC ACG 3’ | Full length cloning of *WsCAS* |
